# Non-invasive flow mapping of parasagittal meningeal lymphatics using 2D interslice flow saturation MRI

**DOI:** 10.1186/s12987-023-00446-z

**Published:** 2023-05-26

**Authors:** Jun-Hee Kim, Roh-Eul Yoo, Seung Hong Choi, Sung-Hong Park

**Affiliations:** 1 Department of Bio and Brain Engineering, Korea Advanced Institute of Science and Technology (KAIST), Daejeon, South Korea; 2 Department of Radiology, Seoul National University College of Medicine, Seoul, South Korea; 3 Department of Radiology, Seoul National University Hospital, Seoul, South Korea

**Keywords:** Meningeal lymphatic vessel, Flow velocity, Parasagittal, Magnetic resonance imaging, Non-invasive, Perfusion imaging, ALADDIN

## Abstract

The clearance pathways of brain waste products in humans are still under debate in part due to the lack of noninvasive imaging techniques for meningeal lymphatic vessels (mLVs). In this study, we propose a new noninvasive mLVs imaging technique based on an inter-slice blood perfusion MRI called alternate ascending/descending directional navigation (ALADDIN). ALADDIN with inversion recovery (IR) at single inversion time of 2300 ms (single-TI IR-ALADDIN) clearly demonstrated parasagittal mLVs around the human superior sagittal sinus (SSS) with better detectability and specificity than the previously suggested noninvasive imaging techniques. While in many studies it has been difficult to detect mLVs and confirm their signal source noninvasively, the detection of mLVs in this study was confirmed by their posterior to anterior flow direction and their velocities and morphological features, which were consistent with those from the literature. In addition, IR-ALADDIN was compared with contrast-enhanced black blood imaging to confirm the detection of mLVs and its similarity. For the quantification of flow velocity of mLVs, IR-ALADDIN was performed at three inversion times of 2000, 2300, and 2600 ms (three-TI IR-ALADDIN) for both a flow phantom and humans. For this preliminary result, the flow velocity of the dorsal mLVs in humans ranged between 2.2 and 2.7 mm/s. Overall, (i) the single-TI IR-ALADDIN can be used as a novel non-invasive method to visualize mLVs in the whole brain with scan time of ~ 17 min and (ii) the multi-TI IR-ALADDIN can be used as a way to quantify the flow velocity of mLVs with a scan time of ~ 10 min (or shorter) in a limited coverage. Accordingly, the suggested approach can be applied to noninvasively studying meningeal lymphatic flows in general and also understanding the clearance pathways of waste production through mLVs in humans, which warrants further investigation.

**Supplementary Information:**

The online version contains supplementary material available at 10.1186/s12987-023-00446-z.

## **Background**

The glymphatic system and meningeal lymphatic vessels (mLVs) provide new insights into the waste clearance of central nervous system (CNS) [1, 2]. Before brain lymphatics were re-studied recently, it was suggested that CNS waste is cleared through multiple pathways [3]. These include the traditional pathway through arachnoid villi and granulations, outflow along the cranial nerves including olfactory route and optic nerve route, and outflow from the spinal nerves [3–6]. Based on the recent studies on the CNS clearance, mLVs are found to be one of the main routes of waste product outflow compared to the other pathways [7, 8]. In addition, recent studies have shown that the mLVs contribute to the outflow process of immune cells, tumor cells or macromolecules into the cervical lymph nodes and that impairment in mLVs are related to neurodegenerative diseases such as Parkinson’s and Alzheimer’s diseases [9–13].

**Fig. 1:**
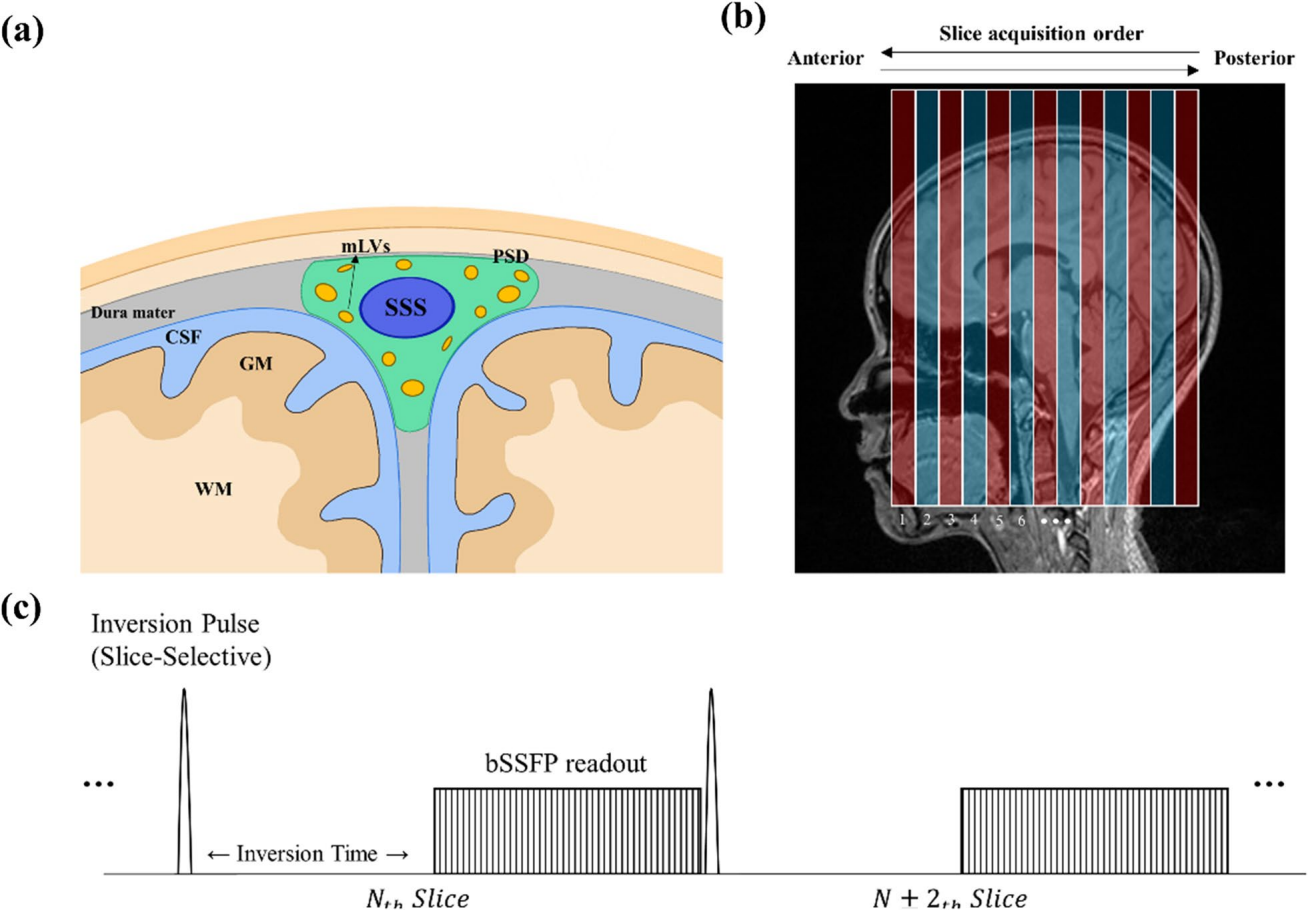
**a** Anatomical structure of parasagittal meningeal lymphatics, **b** IR-ALADDIN imaging scheme and **c** IR-ALADDIN sequence diagram.* IR-ALADDIN* Inversion-recovery alternate ascending/descending directional navigation, *mLVs* Meningeal lymphatic vessels, *CSF* Cerebrospinal fluid, *GM* Gray matter, *WM W*hite matter, *SSS* Superior sagittal sinus, *PSD* Parasagittal dura mater

In-vivo imaging of the brain lymphatics has gained importance due to their functionality in the CNS waste clearance as mentioned above. The meningeal lymphatics are mainly distributed in the dorsal meninges around the superior sagittal sinus (Fig. 1a) and the skull base [7–9, 14–16]. Several studies have used contrast-enhanced MRI to visualize the brain lymphatic system, with intravenous and intrathecal injections being commonly used [9, 14, 16–19]. Intravenously injected molecules move from the blood vessels to the mLVs. Thereafter, black-blood (BB) imaging suppresses the blood signal and emphasizes the lymphatic signal clearly [14, 16, 17]. The intrathecal injection studies directly demonstrated that the contrast agent molecules in the cerebrospinal fluid (CSF) are drained into the lymphatic system [9, 18]. Non-invasive in vivo MR imaging of mLVs have also been performed to reduce the risk of side effects of MRI contrast agent [20]. FLAIR (fluid attenuated inversion recovery) imaging is one of the most common techniques in routine clinical MRI and can also be used to visualize the lymphatic structure of the brain [14, 21]. TOF (time-of-flight) imaging was applied to dorsal lymphatics to show their anatomy and flow direction [22]. From the previous non-invasive imaging studies, the distribution and anatomical structure of the brain lymphatic network were partly demonstrated, but still underexplored due to the small size and slow flow of mLVs. Therefore, brain lymphatic imaging techniques still need to be developed for measurement of noninvasive in-vivo functionality of mLVs in the whole brain in general.

Among the noninvasive MR imaging techniques, arterial spin labeling is a standard method to measure blood perfusion noninvasively. Most ASL methods are dedicated to label fast-moving arterial blood and utilize fast readout sequences based on echo planar imaging or spiral trajectory for measuring perfusion of blood, therefore they may not be appropriate for imaging small and slowly-moving mLVs and measuring their flow. Alternate Ascending/Descending Directional Navigation (ALADDIN) is an ASL technique based on inter-slice labeling effects with no separate labeling plane and utilizes balanced steady state free precession (bSSFP) readout [23, 24]. Because of automatic tracking of the labeling plane to the nearby imaging plane and the high spatial resolution of the bSSFP readout, ALADDIN has the potential to sensitize the flow of mLVs by overcoming their slow flow and small size.

In this study, we propose inversion-recovery ALADDIN (IR-ALADDIN) as a new imaging method of mLVs. ALADDIN may be optimized for sensitizing the slow flow of the small mLVs, and IR may enable us to distinguish fluids in mLVs from those in other structures. Also IR-ALADDIN at multiple inversion times (TIs) may enable us to quantify flow velocity of mLVs in humans. The proposed IR-ALADDIN technique was tested in both human parasagittal meningeal lymphatics and a flow phantom with variable flow velocities. The anatomical structure and flow of the lymphatic system measured with IR-ALADDIN were compared with those from the contrast-enhanced black blood imaging technique and the literature. A simulation study was performed to predict how the IR-ALADDIN signal changes as a function of a few scan parameters. Potential applications of the proposed method for studying neurodegenerative diseases are discussed.

## **Methods**

### **IR-ALADDIN sequence**

We mainly used ALADDIN for imaging mLVs in this study. The ALADDIN technique was introduced for the acquisition of perfusion-weighted imaging based on the inter-slice blood saturation effects where labeling planes automatically track the imaging plane with no separate labeling/control preparation. ALADDIN acquires imaging slices in alternating slice orders (ascending/descending), thus it provides information on flow direction and perfusion (Fig. 1b). More importantly, ALADDIN uses prior imaging slices as labeling plane. Because of the short distance between labeling and imaging slices, it has high sensitivity to slow flow components like lymphatics. To emphasize human meningeal lymphatic vessels (mLVs) in this study, we added a slice-selective inversion recovery pulse to the ALADDIN (IR-ALADDIN) for each slice (Fg.1c) with an inversion time (TI) of 2300 ms. The inversion pulse suppressed the surrounding CSF signal which could be potentially misunderstood as mLVs (Fig. 1a).

### **Simulation of the IR-ALADDIN signal**

The IR-ALADDIN signal was simulated with varying TI values and flow velocities. Based on the Bloch equation and bSSFP physics, the signal intensity of bSSFP ALADDIN image could be calculated as below [25, 26].

The spin magnetization vector $\vec{M} = (M_{x} ,M_{y} ,M_{z} )^{T}$                 

Bloch equation, $$\frac{{d\vec{M}}}{{dt}} = \gamma \left( {\vec{M} \times \vec{B}} \right) - \left( {\begin{array}{*{20}c} {\frac{{M_{x} }}{{T_{2} }}} \\ {\frac{{M_{y} }}{{T_{2} }}} \\ {\frac{{M_{z} - M_{0} }}{{T_{1} }}} \\ \end{array} } \right)$$ 

where ${M}_{0}$ is the equilibrium spin magnetization, ${T}_{1},{T}_{2}$ are spin relaxation times and *γ* is the gyromagnetic ratio.

Then the signal intensity at time point, t, could be obtained from summation of the local magnetizations.

$$S\left( t \right) = \mathop \sum \limits_{r} \vec{M}\left( {r,t} \right)\vec{x} + j\mathop \sum \limits_{r} \vec{M}\left( {r,t} \right)\vec{y}$$

Both ascending and descending ALADDIN imaging was simulated based on the bSSFP with a train of magnetizations, which were calculated for each repetition-time (TR) period. Imaging parameters applied for the simulation were slice thickness = 5 mm, TR = 4.84 ms, echo time (TE) = 2.42 ms, slice gap = 100%, and flip angle = 60°. We simulated three IR-ALADDIN slices, where two lateral slices worked as labeling slices of the center slice.

Signals from various parasagittal compartments were simulated with simulation parameters for each compartment as follows. Venous blood : T1 = 1584 ms [27], T2 = 154 ms [28], flow velocity = 13 cm/s [29]; lymphatic fluid : T1 = 3100 ms [30], T2 = 610 ms [30], flow velocity $\cong$1 mm/s [31–33]; parietal cerebral subarachnoid CSF : T1 = 4000 ms [34, 35], T2 = 2030 ms [36], net flow velocity$\cong$ 0 mm/s [37–39]; Arteriole : T1 = 1664 ms [27], T2 = 154 ms [28], pial arteriole flow velocity = 10 mm/s [40]. We assumed all the flows to be laminar flows.

### **Flow phantom experiment**

**Fig. 2:**
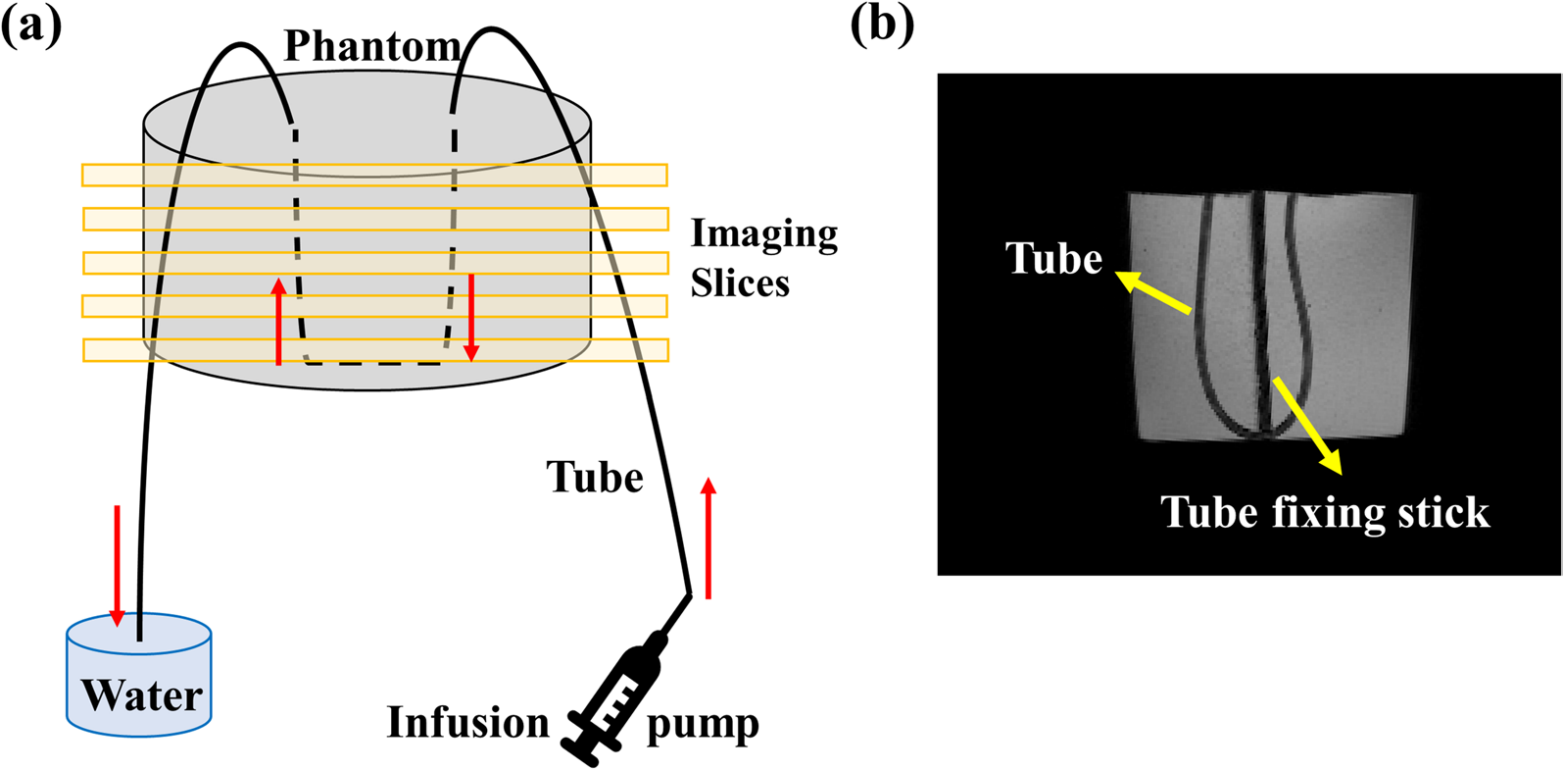
Flow phantom setting scheme,** a** Diagram of flow phantom, infusion pump and coronal IR-ALADDIN slices. Water flows into the tube from the infusion pump and flows out to the water tank outside of MRI room. **b** Minimum intensity projection of phantom 3D T1 images

A flow phantom experiment was conducted to confirm the flow rate estimation from IR-ALADDIN PSC images. The flow phantom was prepared with a 4% agarose phantom and a penetrating tubing (ACF00002-F) filled with water (Fig. 2). An infusion pump (KdScientific Legato 110) was used to generate the controlled flow rate through the tubing. Based on the inner diameter of the tube, we could regulate the flow velocity of fluid in the tubing by modulating infusion rate of the infusion pump. We tested flow velocity from 0 mm/s to 8 mm/s.

### **Participants and approvals**

All the experiments were performed on a 3T whole-body scanner (Trio, Siemens Medical Solutions, Erlangen, Germany) with a body coil transmission and a head coil reception. This study was approved by Institutional Review Board and written informed consent was obtained before the experiment. The total number of volunteers was 28. All of them participated in the IR-ALADDIN experiment, 8 of them underwent the IR-ALADDIN scan twice for repeatability check, 16 of them participated in the CE-BB imaging, 1 of them participated in the FLAIR and TOF imaging, and 4 of them participated in the multi-TI IR-ALADDIN experiment.

### **MRI acquisition**

#### **IR-ALADDIN acquisition**

For the human experiment, the IR-ALADDIN bSSFP imaging parameters were TR/TE = 4.84/2.42 msec, flip angle = 60°, matrix size = 256 × 256, field-of- view (FOV) = 250 × 250  mm^2^, thickness = 5 mm, gap = 5 mm (100% of thickness), scan direction = coronal, PE order = centric, slice-selective TI = 2300 ms, and PE direction = left–right. Each slice took 3.539 s for acquisition. Ascending/descending directional full sets (8 measurements) were acquired with number of slices = 19 and then another two full sets (8 measurements) were acquired with number of slices = 18. The former and the latter covered the whole brain without gaps with total scan time = 17 min 27 s. For the subjects with CE–BB imaging, IR-ALADDIN was acquired with number of slices = 9 and total scan time = 4 min 15 s. For the flow phantom experiment, IR-ALADDIN was acquired with the same scan conditions as the human experiment except number of slices = 7, inversion times = 2000 ms, 2300 ms, 2600 ms, and total scan time = ~ 10 min. Lastly, we performed preliminary in-vivo study with multi-TI IR-ALADDIN on 4 healthy volunteers, and the imaging parameters were the same as the multi-TI phantom experiment. Thus, the total scan time for in-vivo multi-TI IR-ALADDIN was about 10 min for each subject.

#### **Black-blood imaging acquisition**

A contrast-enhanced 3D T1 black-blood images were acquired as a comparison reference to IR-ALADDIN. 3D T1 black-blood imaging parameters were TR/TE = 620/15msec, flip angle = variable, matrix size = 256 × 256, FOV = 250 × 250 mm^2^, thickness = 1.2 mm, scan direction = sagittal, number of averages = 1, echo train length = 21 and total scan time = 5 min 35 s. 3D T1 black-blood imaging was performed before and after contrast agent i.v. injection. We used Gadovist as a contrast agent with a high tendency to extravasate through a permeable endothelial barrier [14].

#### **Time-of-flight imaging and FLAIR acquisition**

As a comparison reference to IR-ALADDIN, fluid-attenuated inversion recovery (FLAIR) images and time-of-flight (TOF) MR angiogram were additionally acquired [14, 21, 22]. For both FLAIR and TOF MRA, FOV and imaging slice positions were identical to those of IR-ALADDIN. The imaging parameters for FLAIR were TR/TE = 8000 / 92 msec, TI = 2300 ms, echo train length = 17, concatenation = 1, flip angle = 160°, matrix size = 768 × 476, FOV = 250 × 250 mm^2^, thickness = 4 mm, gap = 6 mm (150% of thickness), number of slices = 11 for odd and 10 for even, acquisition bandwidth = 130 Hz/pixel, scan direction = coronal, PE direction = left–right, and total scan time = 2 min 24 s. The imaging parameters for TOF MRA were TR/TE = 35/ 5 msec, flip angle = 10°, matrix size = 448 × 448, FOV = 250 × 250 mm^2^, thickness = 2 mm, saturation band-imaging slice gap = 5 mm, saturation band thickness = 20 mm, number of slices = 1, average = 10, acquisition bandwidth = 319 Hz/pixel, scan direction = coronal, PE direction = left–right, and total scan time = 1 min 24 s. TOF images were acquired with three different saturation bands of (i) posterior saturation band only, (ii) anterior saturation band only, and (iii) both, all of which took 4.2 min.

### **Data processing**

All the data analyses were performed using Matlab R2020a (Mathworks, Natick, MA). For IR-ALADDIN data processing, four ascending (Asc) and four descending (Dsc) acquisitions were averaged separately and then subtracted from each other to maximize the flow signals, which have directionality. To visualize the mLVs better, the images were displayed as subtraction images (Asc-Dsc) as well as percent signal changes (PSC) (Asc-Dsc)/S*100, where Asc and Dsc represent the averaged ascending and descending images and S represents the average of Asc and Dsc. The ROIs of IR-ALADDIN mLVs adjacent to the SSS were manually segmented based on its signal enhancement in the IR-ALADDIN PSC images. Furthermore, we processed IR-ALADDIN brain tissue, mLVs, and SSS surface mesh by using ITK-SNAP [41]. The segmented surface mesh data were overlaid and built up to a 3D brain model using Para-View (Kitware). To evaluate the characteristics of mLVs in comparison with other brain compartments, ROIs of gray matter (GM), the SSS and CSF were manually determined based on their anatomy in the IR-ALADDIN baseline images and then the average of PSC values from each ROI was analyzed for comparison.

### **Comparison between IR-ALADDIN and black-blood imaging**

IR-ALADDIN 2D multi-slice data and CE-BB 3D data were registered using SimpleITK [42]. To compare the similarity of the mLVs ROIs between IR-ALADDIN and CE-BB images, we paired each IR-ALADDIN slice with a CE-BB coronal slice in the same position. The mLVs signal enhancement from CE-BB imaging was detected by comparing the pre-injection and post-injection images. Unlike IR-ALADDIN which has a semi-quantitative PSC value for comparison, the BB image only provides anatomical information of the mLVs based on the signal enhancement with contrast agent. Therefore, the morphological similarity in mLVs between CE-BB subtracted image (i.e., post−pre) and IR-ALADDIN PSC/subtracted image was demonstrated visually.

## **Results**

### **Simulation results of the IR-ALADDIN**

**Fig. 3:**
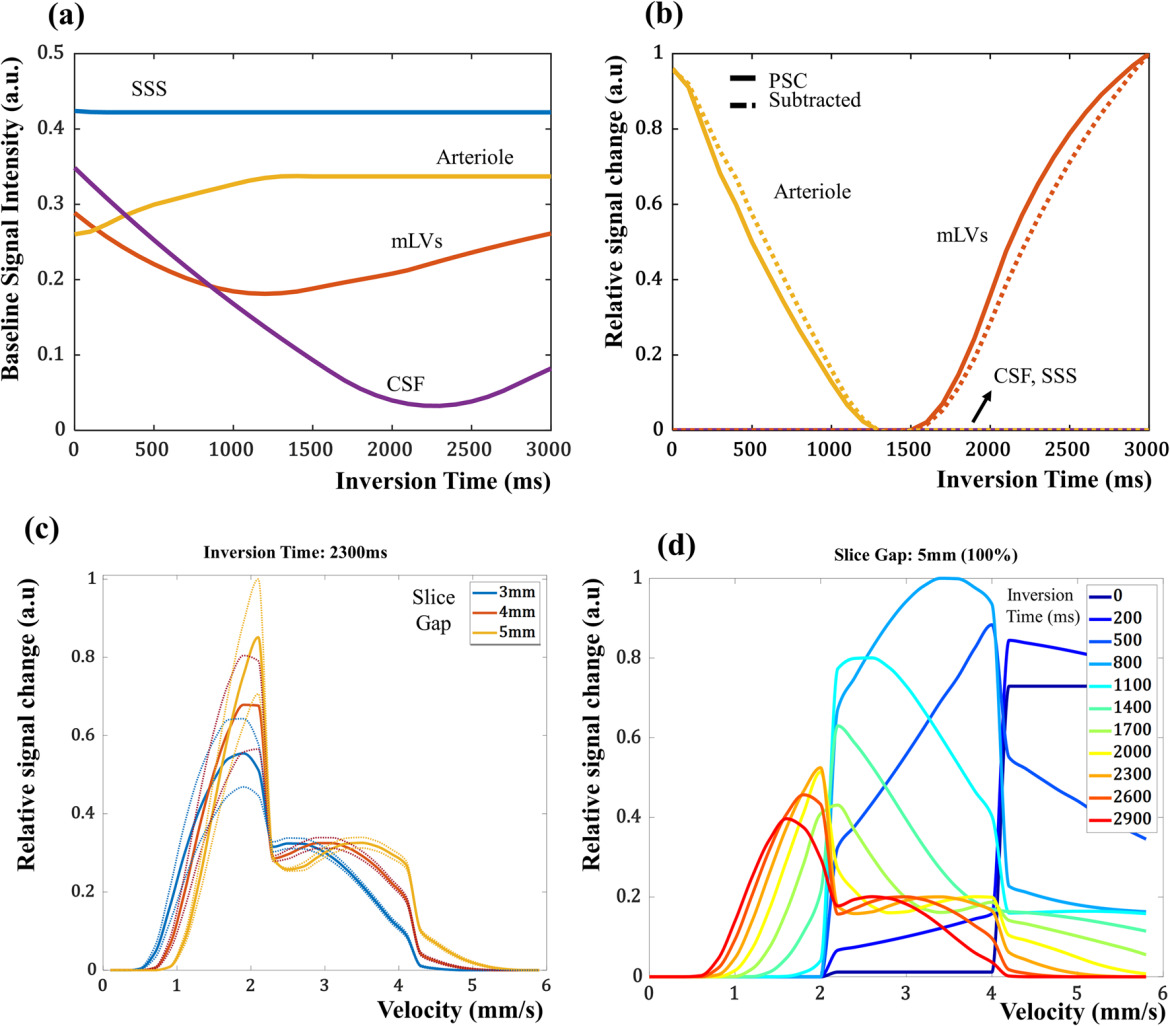
IR-ALADDIN signal intensity simulation. Simulation results with varying inversion time for different tissues, **a** plot of baseline signal intensity, **b** plot of relative signal changes from PSC and subtraction. Simulation results of relative percent signal changes of lymphatic flow with **c** varying slice gap (dotted line: relaxation time varies − 10–10%) and **d** varying inversion time. Relative signal change: relative ratio of percent signal changes. mLVs : meningeal lymphatic vessels with flow velocity of 1 mm/s for (**a**) and (**b**), *CSF* Cerebrospinal fluid with net flow velocity of 0 mm/s, *SSS* Superior sagittal sinus with flow velocity of 13 cm/s, *Arteriole* Arteriole with flow velocity of 10 mm/s

We simulated potential flow compartments around the human parasagittal area, which were SSS, arteriole, CSF and mLVs. The simulation result predicted that TI of 2300ms achieved the best contrast between lymphatics and the other compartments and also well suppressed CSF signal in the IR-ALADDIN baseline images (Fig. 3a). As expected, arteriole and lymphatics had signals in PSC and subtraction images, and only lymphatics had signal when TI = 2300 ms (Fig. 3b). It is expected for IR-ALADDIN to capture the slow mLVs flow (order of 1 mm/s), but it would be hard for IR-ALADDIN to capture the fast flows such as arterioles (10 mm/s, pial arteriole [40]) (Fig. 3c). Based on the IR-ALADDIN imaging setting (Fig. 1), the fastest measurable flow velocity should be (gap + thickness)/inversion_time = 10 mm/2300 ms = 4.34 mm/s theoretically. Any flows exceeding this flow velocity will have no labeling difference between ascending and descending acquisitions, and thus the flow information cannot be detected in the subtraction or PSC images. The slice gap of 5 mm was used for IR-ALADDIN acquisition to maintain sensitivity of slow flow like mLVs and also to avoid crosstalk effects. Lastly, we simulated various inversion times for IR-ALADDIN mLVs imaging. The simulation result shows that using an inversion time of 2300 ms or longer would be suitable to detect flow velocity of 1 mm/s (Fig. 3d). For the selection of the inversion time, we also considered increasing SNR and excluding the CSF signals. A shorter TI would allow higher SNR due to shorter signal relaxation, and TI = 2300 ms was the best for excluding the CSF signal by suppression. Overall the simulation predicted that only mLVs flow signals would remain in the IR-ALADDIN with TI of 2300 ms. Thus, we used TI = 2300 ms for IR-ALADDIN in the real experiments.

### **Flow phantom imaging result of the IR-ALADDIN**

**Fig. 4:**
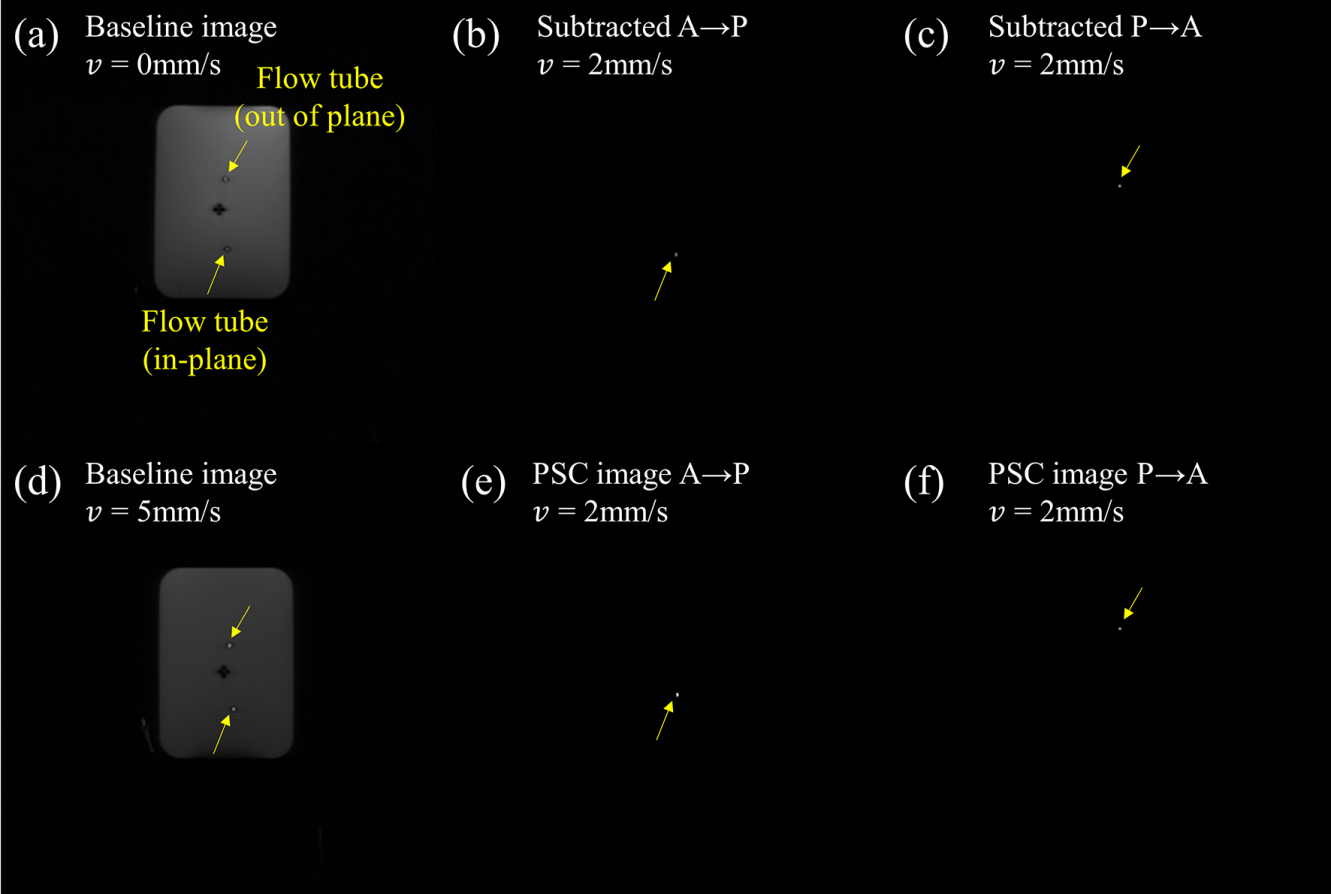
IR-ALADDIN images of flow phantom.** a**, **d** Baseline images of flow phantom, **b**, **c** subtracted images at flow velocity 1.3 mm/s, **e**, **f** PSC images at flow velocity 1.3 mm/s

**Fig. 5:**
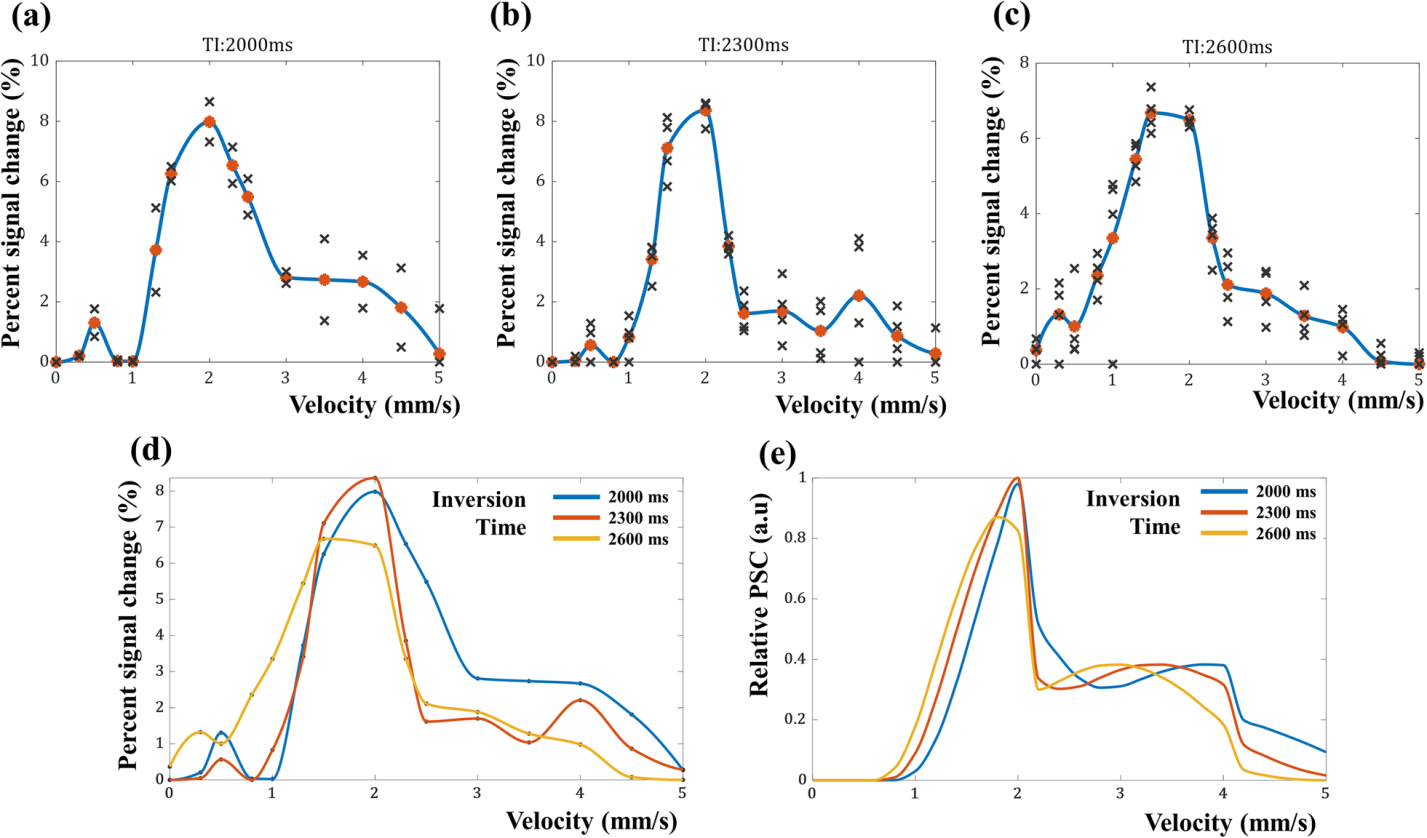
IR-ALADDIN PSC results of the flow phantom experiment. PSC as a function of flow velocity measured with inversion time of **a** 2000 ms, **b** 2300 ms, and **c** 2600 ms, of the in-plane tube. Black cross points represent raw PSC signal from multiple slices. Orange dots represent average PSC from multiple slices. **d** PSCs as a function of flow velocity measured at all inversion times. **e** IR-ALADDIN signal intensity simulation at different inversion times. PSC: percent signal change

After IR-ALADDIN acquisition, the same IR-ALADDIN image processing steps were applied to demonstrate PSC images from the flow phantom data. The PSC of flow tube was acquired from the in-plane tube ROI (Fig. 4), because the water in the out-plane tube might be affected by labeling effects from the in-plane tube in an unintended way. From the in-plane tube results, the relation between the flow velocity and the PSC could be demonstrated (Fig. 5). The flow phantom experiment would reflect the real imaging environment of IR-ALADDIN better than the simulation. Thus we used the flow phantom results to infer the flow velocity from the PSC results of IR-ALADDIN in vivo. Furthermore, by varying the inversion time, the PSC curve of IR-ALADDIN shifted to the lower velocity as inversion time decreased from 2300ms to 2000ms, vice versa when it increased from 2300 ms to 2600 ms (Fig. 5d). The measured phantom PSC curves from different inversion times were found to be similar to the simulated IR-ALADDIN relative PSC results (Fig. 5e). Also, this result in Fig. 5d, e is somehow consistent with our expectation that in the lower velocity range (uphill) the labeled mLV has not fully reached the imaging slice and thus a longer time (TI = 2600ms) will increase the signals. Conversely, in the higher velocity range (downhill), the opposite is expected. Multi TI IR-ALADDIN could specify the flow velocity by monitoring the changes in the measured PSC values at different TIs.

### **Imaging parasagittal mLVs by IR-ALADDIN**

**Fig. 6:**
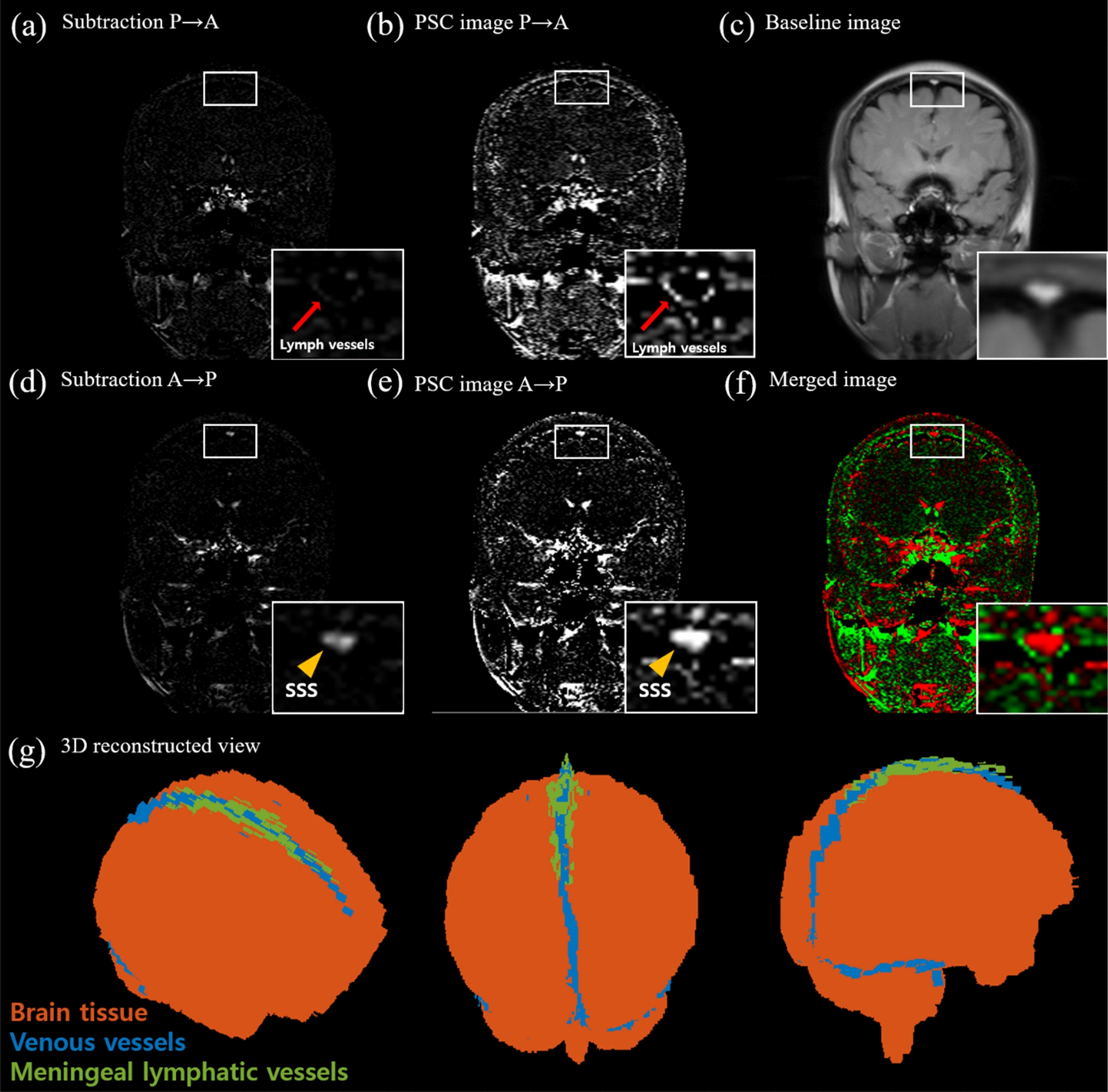
Representative image of IR-ALADDIN. **a**,** d** Subtraction images, **b**–**e** PSC images, **c** Anatomical baseline image, **f** Merged bidirectional image (anterior to posterior and posterior to anterior), **g** 3D reconstructed IR-ALADDIN with brain tissue (orange), venous vessels (blue) and meningeal lymphatic vessels (green). *PSC* Percent signal change

The subtraction and PSC images of IR-ALADDIN showed high blood flow signals inside the SSS in the anterior to posterior direction (A→P), consistent with the direction of venous blood flow in the SSS (Fig. 6d, e). Also high flow signals were found outside but adjacent to the SSS in the posterior to anterior direction (P→A), where dorsal mLVs are mainly found (Fig. 6a, b) [22]. The CSF signals typically detected as the brightest pixels in the bSSFP were completely suppressed in the baseline bSSFP images in this study because of the IR pulse (Fig. 6c). The mLVs were observed in multiple slices of IR-ALADDIN (Additional file 1: Fig. S1) in all the tested subjects (Additional file 1: Fig. S2). The visibility of mLVs was variable across slices due to the large structural and flow-related variability depending on the location of mLVs (Additional file 1: Fig. S1). Anatomical locations of mLVs detected in IR-ALADDIN were consistent with those detected in the previous studies, like parasagittal dura of FLAIR, CE-BB, and TOF (Figs. 6f, 8 and 9) [14, 16, 17, 21, 22]. Furthermore, 3D reconstructed IR-ALADDIN demonstrated the distribution of the mLVs which flows around the SSS (Fig. 6g). Lastly, to evaluate repeatability of IR-ALADDIN mLVs detection, we acquired two sets of IR-ALADDIN in the same subjects with identical imaging settings. The detection of mLVs from both test-retest IR-ALADDIN were confirmed from its signal enhancement adjacent to the SSS (Additional file 1: Fig. S3).

### **PSC measurement of parasagittal lymphatic vessels from single-TI IR-ALADDIN**

**Fig. 7:**
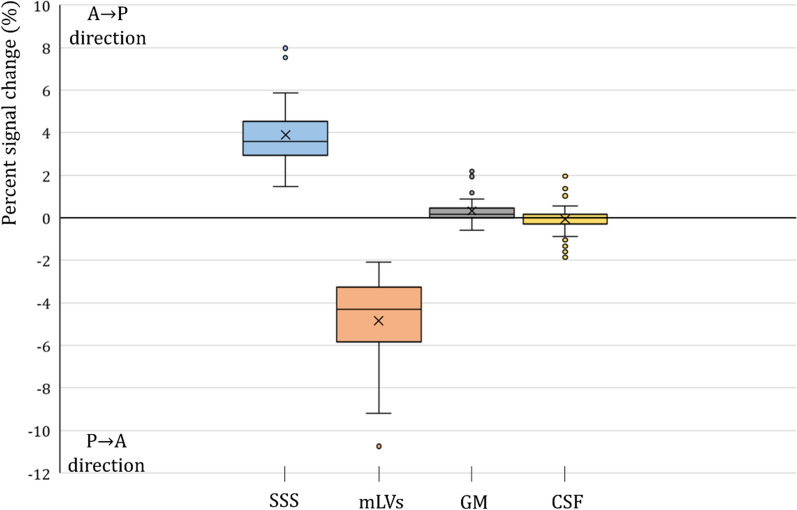
The box plot of percent signal change (PSC) of SSS, mLVs, GM and CSF from the PSC image. Positive PSC represents the A→P direction and negative PSC represents the P→A direction of IR-ALADDIN images. Cross points represent the mean PSC of each tissue, and outliers were pointed in circle outside of the boxes. *SSS* Superior sagittal sinus, *mLVs M*eningeal lymphatic vessels, *GM* Gray matter, *CSF* Cerebrospinal fluid

The averaged PSC values from the CSF, the SSS, GM, and mLVs ROIs of IR-ALADDIN are shown in Fig. 7. Each anatomical region had different PSC distributions, supporting the notion that the dorsal mLVs signals in the IR-ALADDIN are not from CSF, the SSS, or brain tissue (GM) (Fig. 7).

From the flow-phantom result of IR-ALADDIN (Fig. 5b) and the averaged PSC values of mLVs (Fig. 7), we could roughly estimate the flow velocity of mLVs in vivo. The mean PSC of mLVs from the IR-ALADDIN ROIs was 4.84 ± 1.99 (%) and the upper-lower quartile range was −3.2 to −5.8 (%). These PSC values of mLVs might correspond to either the lower velocity range (uphill) or the higher velocity range (downhill) (Figs. 5b and 7). This uncertain range could be clarified by multi-TI IR-ALADDIN as explained below.

### **PSC measurement of parasagittal lymphatic vessels from multi-TI IR-ALADDIN**

**Fig. 8:**
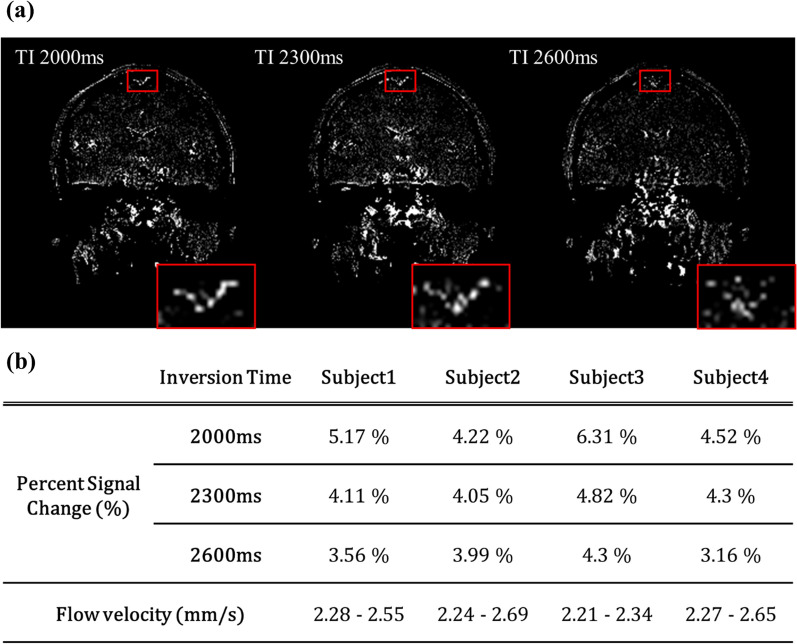
Preliminary results of in-vivo multi TI IR-ALADDIN experiment.** a** P→A directional PSC images from different inversion times. Red boxes represent the region of parasagittal mLVs. **b** Averaged PSCs from different inversion times and 4 healthy subjects. *PSC* Percent signal change (%)

After preprocessing the IR-ALADDIN data including image registration between those from multiple TIs, mLVs ROI was determined from PSC images based on the morphology and signal intensity (Fig. 8a). For all the tested subjects, the PSC from TI 2000 ms was higher than the PSC from TI 2300 ms and TI 2600 ms, which indicates that the measured flow velocity of mLVs corresponded to the higher velocity range (downhill) over the 2 mm/s (Fig. 5). Based on the measured PSCs, we estimated preliminary in-vivo mLVs flow velocities, which were distributed between the range of 2.2–2.7 mm/s (Figs. 5 and 8b).

### **Comparison of parasagittal mLVs imaging techniques**

**Fig. 9:**
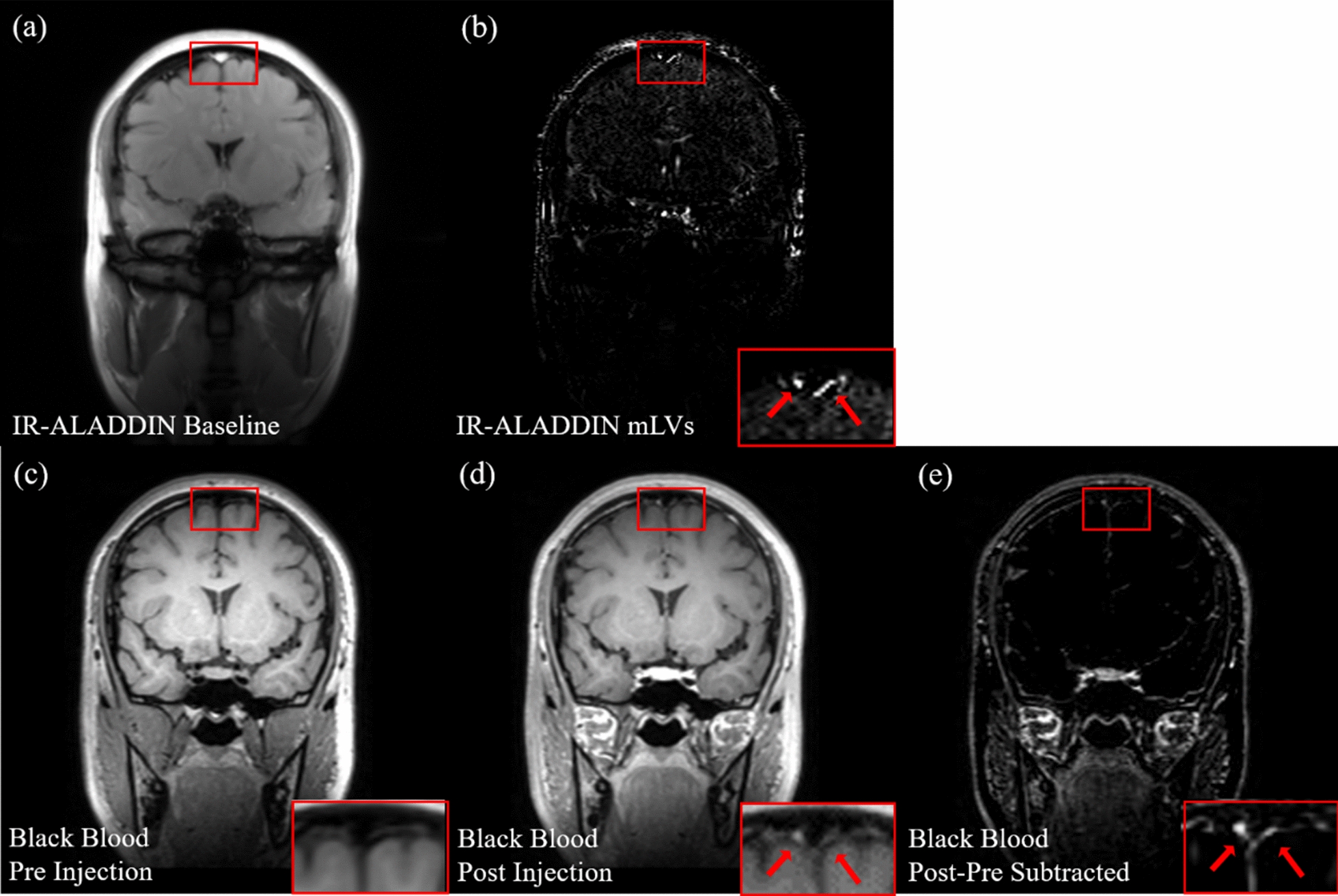
Representative images for visual comparison between IR-ALADDIN mLVs image and contrast-enhanced black blood mLVs images with pre/post injection. **a** IR-ALADDIN baseline image, **b** mLVs image from IR-ALADDIN with flow direction of P→A, **c**, **d** Black blood images with pre and post injection, **e** subtracted image between post and pre-injection black blood images. Red arrows represent brightened mLVs structures. mLVs : meningeal lymphatic vessels

**Fig. 10:**
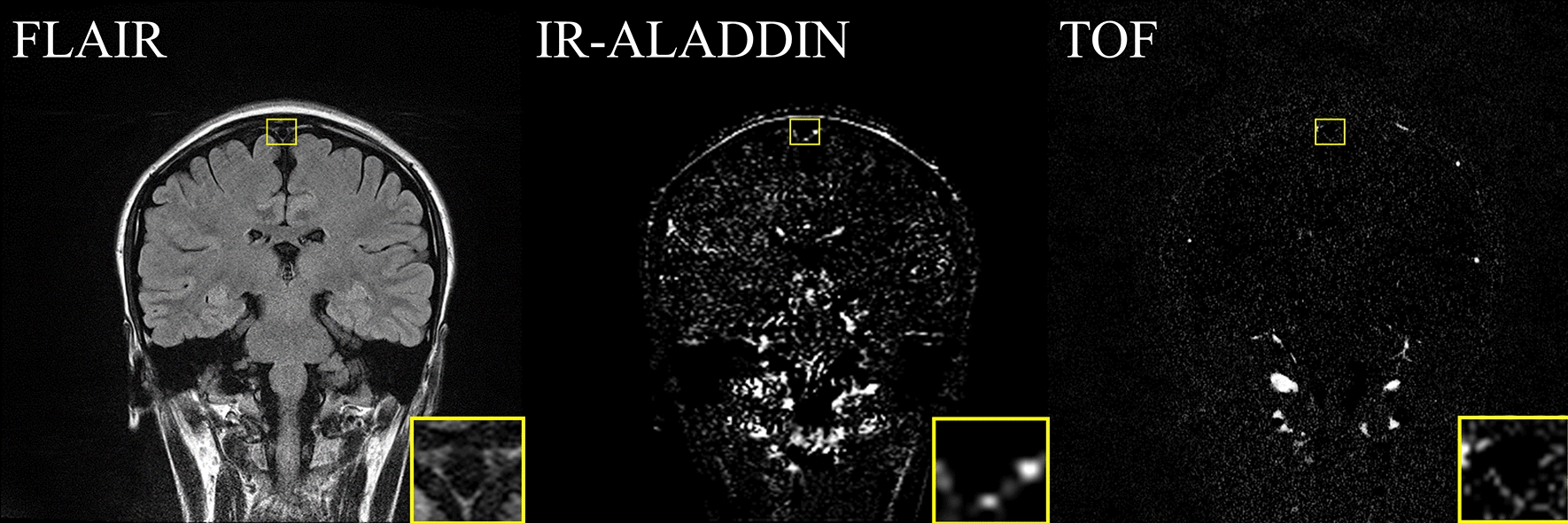
Comparison of mLVs images between FLAIR, IR-ALADDIN, and TOF in the same imaging position. Yellow boxes indicate the location of mLVs around the SSS

Black blood imaging with intravenous contrast injection shows elevated mLVs signals around SSS. By comparing pre- and post-injection BB images, the brightened mLVs were confirmed (Fig. 9c–e). The anatomical structure and location of mLVs from IR-ALADDIN were similar to those of mLVs from the BB imaging (Fig. 9a, b and Additional file 1: Figs. S4–S8). In addition, we compared the proposed IR-ALADDIN with other noninvasive mLVs imaging techniques, FLAIR and TOF. Based on the TOF sequence settings, the TOF was able to capture flow in the velocity range between 0.6 mm/s and 3.26 mm/s. The TOF showed weak mLVs signals due to its low SNR (Fig. 10). FLAIR revealed an area of parasagittal dura mater, which included the area of mLVs from IR-ALADDIN but was less specific to vascular structure (mLVs) compared to IR-ALADDIN (Fig. 10).

## **Discussion**

The flow mapping of mLVs has not been easy because of their small size and slow flow, requiring high spatial resolution and high sensitivity to slow flow. Fluid velocity in mLVs is too slow to be measured with even the phase contrast MRI, a standard imaging method for mapping CSF flow. ALADDIN, which is based on interslice saturation effects and the high resolution bSSFP readout, could detect the slow flow of the small mLVs across the tested subjects (Fig. 6 and Additional file 1: Figs. S2, S4–S8). Inversion recovery was effectively used to emphasize signals of mLVs while suppressing those of CSF (Figs. 3 and 6). While it was applied to detect parasagittal meningeal lymphatic vessels in this study, it can be applied to the whole brain in various directions depending on mLVs of interest, because of the automatic tracking of the labeling planes (prior slices) to the nearby imaging plane. Detection of mLVs in other brain regions is beyond of the scope of the current study.

Multiple scans of IR-ALADDIN with different TIs could confirm a specified velocity range (Fig. 5d, e), suggesting that the flow velocity of mLVs in humans was in the range of 2.2−2.7 mm/s. If the mLVs flow velocity corresponded to the lower velocity range < 2 mm/s (uphill), then the measured PSC of mLVs would have increased at TI 2600 ms and decreased at TI 2000 ms, compared to that measured at TI 2300 ms (Fig. 5d, e). Thus, when the purpose of the experiment is not for mapping mLVs structure but for measuring flow velocity, then multi TI IR-ALADDIN with a smaller number of slices can be applied. In case of 7 slices of IR-ALADDIN positioned to cover mLVs, acquisition of three TI IR-ALADDIN takes about 10 min. The multi-TI phantom experiment also suggests that for mapping the PSC value to the velocity of mLVs, TI of 2600 ms is advantageous in the lower velocity range (uphill) and TI of 2000 ms is advantageous in the higher velocity range (downhill) because of their less steep slopes. In this sense, only the two TI values of 2000 and 2600 ms (rather than the three-TI values) can potentially be used to estimate the velocity, which can reduce the scan time even further.

So far, the flow velocity of meningeal lymphatic vessels has not been reported in humans. Previous in-vivo studies have reported lymphatic flow in human skin or breast lymphatics to be around 1 mm/s [31–33], and thus the velocity of human mLVs measured with IR-ALADDIN in this study (2.2−2.7 mm/s) is slightly higher but roughly consistent with previous studies in terms of scale. It should be noted that the previous human lymphatic flow measurements were conducted in lymphatics close to the skin. Therefore, the slightly higher flow velocity of meningeal lymphatic vessels measured in this study might be reasonable to some degree. Further future studies on mLVs flow velocity in humans are needed to improve the flow velocity prediction from IR-ALADDIN.

While it has been difficult to confirm the detection of mLVs in many in vivo studies, we postulate that the mLVs detected in this study are indeed mLVs for several reasons. First, we could assume that the mLVs signals are not from CSF because the T1 of lymphatic vessels (3100 msec) is different from that of CSF (4000 msec) and the inversion time (2300 ms) was adjusted for the CSF signals to be nulled, which was confirmed in the baseline bSSFP images (Fig. 6).

Second, the meningeal lymphatic vessel structure contains intraluminal valve structure which prevents lymph backflow and thus stimulates unidirectional flow [12]. IR-ALADDIN mLVs around the SSS showed the posterior-to-anterior flow direction, which was opposite to the venous flow direction in the SSS including minor veins merging to SSS. Despite the same flow direction with mLVs, arteriole flow could be separated based on its flow velocity, which is much higher than that of mLVs (Fig. 3b). Also the flow direction of mLVs from IR-ALADDIN was in agreement with the flow direction of dorsal mLVs reported in a previous study (Fig. 6) [22].

Third, we could estimate the flow velocity of mLVs from their PSC values by using the PSC-flow velocity relation based on the flow-phantom result (Figs. 5, 7 and 8). This velocity range of human mLVs in this study was slightly higher but on a similar scale to that of lymphatics in human skin or breast lymphatics in the previous studies, as mentioned above.

Fourth, based on previous morphological studies, there were tubular-shaped lymphatic vessels on the periphery of the SSS and some mLVs were distributed around the SSS through the dura mater [15]. The morphological shape and distribution of mLVs from the previous studies were consistent with those of the high flow signals around the SSS in this study (Fig. 6). Also, we confirmed that the PSC distributions of mLVs were completely different from those of nearby other structures such as CSF, the SSS, and GM (Fig. 7). The PSC of the IR-ALADDIN mLVs was within a certain range (lower quartile 3.2% to upper quartile 5.8%), indicating that IR-ALADDIN steadily shows meningeal lymphatic flows (Fig. 7).

Finally, we compared the proposed mLV imaging technique, IR-ALADDIN, with the existing mLV imaging technique, the contrast-enhanced BB imaging technique [14, 16, 17]. From the visual morphology perspective, it was confirmed that there was reasonable agreement in the mLVs between IR-ALADDIN and contrast-enhanced BB (Fig. 9 and Additional file 1: Figs. S4–S8). Based on all of these factors, we believe that the high flow signals around the SSS in this study were from dorsal parasagittal mLVs.

There are a few things to be noted and to be improved in future studies. First of all, we utilized the IR-ALADDIN simulation to explain our imaging results. The simulation was conducted based on the laminar flow assumption, but most human lymphatic vessels flow in a pulsatile manner. This flow movement difference between simulation and in-vivo could result in estimation error of PSC or flow velocity. However, still in-vivo mLVs flow movement has not been studied, thus we tried to estimate the mLVs velocity under the assumption of laminar flow. Although the flow phantom might not perfectly mimic the in vivo conditions, we could estimate the range of mLV flow velocities through the phantom study and in-vivo IRALADDIN data. This will be helpful for various studies related to mLVs.

We described the scan time of IR-ALADDIN for the whole brain coverage. In this case, two groups of imaging protocols (odd, even slices) were acquired with 19 and 18 slices for each. It took total 17 min to acquire all of them. In terms of scan time acceleration, conventional imaging acceleration techniques such as SENSE, GRAPPA, compressed sensing and deep learning can be applied. However, it would go beyond the scope of this study. Nonetheless, if the study purpose is for checking the mLVs flow in each subject instead of demonstrating the detailed anatomy of mLVs in the whole brain, it may be possible to acquire only a single group with smaller number of slices (e.g. 9 slices) with 4.25 min, as also demonstrated in the multi-TI IR-ALADDIN (7 slices) in this study. Compared to the other flow imaging technique (TOF which takes about 4.2 min only for a single slice), IR-ALADDIN is more efficient.

In addition, IR-ALADDIN PSC images and subtracted images demonstrated SSS flow signals (Fig. 6d, e), even though the simulation results predicted no flow signal from the SSS (Fig. 3b). IR-ALADDIN flow signal was labeled in the prior slices and imaged in the imaging slice with its vessel structure. Thus, the IR-ALADDIN flow image demonstrates structural information of the corresponding slice, and contains information of the flow from the previously labeled slice to the imaging slice. We postulate that the SSS flow signals from the IR-ALADDIN PSC were not from the main stream of SSS, but from the slowly-moving cerebral venous flow merging into the SSS, which was labeled in the brain tissue or small veins [43]. Furthermore, the proposed IR-ALADDIN was compared with the contrast-enhanced black blood imaging technique as reference. While CE-BB imaging technique showed the structural information based on the contrast agent displacement into the lymphatic vessels, IR-ALADDIN imaging technique showed the signal enhancement based on the flow. Any lymphatic vessels in parasagittal dura mater that flow randomly (not unidirectional) or in a direction perpendicular to the A-P or P-A direction, might be captured in CE-BB imaging but not in the IR-ALADDIN. Therefore, mLVs in IR-ALADDIN could be different from those in the CE-BB image, and might be more sensitive to the lymphatic flow information.

Lastly, in this study we suggest (i) single-TI IR-ALADDIN as a novel non-invasive method to visualize mLVs in the whole brain with scan time of ~ 17 min and (ii) the three-TI IR-ALADDIN as a way to quantify the flow velocity of mLVs with a reduced scan time of ~ 10 min (or shorter with two-TI IR-ALADDIN) in a limited coverage. We demonstrated the potential feasibility of measuring the flow velocity of human mLVs noninvasively. However, the quantitative measurement of flow velocity of mLVs needs more optimization and additional studies with a larger number of data.

The IR-ALADDIN imaging technique demonstrated not only the morphology of mLVs, but also the flow direction, PSC and velocity of mLVs around the SSS without using a contrast agent. Because of the noninvasiveness, high resolution, sensitivity to slow flow, and availability of flow directional information, the proposed IR-ALAADIN approach can be helpful for studying brain clearance pathway and applied for studying lymphatic vessels in many human neurological diseases such as Alzheimer’s disease and multiple sclerosis.

## **Conclusion**

In this study, IR-ALADDIN, based on interslice saturation effects and high resolution bSSFP readout, is proposed to detect slow flow of parasagittal meningeal lymphatic vessels in the human brain noninvasively. The proposed IR-ALADDIN detected the mLVs in all the tested subjects, which could be confirmed based on morphological shape and distribution (black blood imaging and literature), flow direction, PSC distributions, the flow phantom experiment and simulations, with better sensitivity and specificity than the previously suggested noninvasive methods. The three-TI IR-ALADDIN enabled us to estimate the flow velocity of human mLVs, which was slightly higher but on a similar scale to the flow velocity of lymphatics in human skin or breast lymphatics reported in the previous studies. Accordingly, the suggested approach can be applied to noninvasively studying meningeal lymphatic flows in general and also understanding the clearance pathways of waste production through mLVs in humans, which warrants further investigation.

## Supplementary Information

**Additional file 1: Figure S1.** IR-ALADDINsubtraction and baseline images across multiple slices from one representativevolunteer. Yellow arrowheads represent superior sagittalsinusand red arrows represent the meningeal lymphatic vessels. **Figure S2.** IR-ALADDINsubtraction images and baseline images from normal volunteers.Yellow arrowheads represent superior sagittal sinusand red arrowsrepresent the meningeal lymphatic vessels. **Figure S3.** IR-ALADDINimages of multiple measurements from the same subject to check therepeatability of IR-ALADDIN. Each red box is enlarged to the white box. Redboxes contain SSS in the A→Pdirection image and mLVs in the P→Adirection image. **Figure S4.** The visualcomparison between contrast-enhanced black blood imaging and IR-ALADDINimaging**.** The mLVs are boxed in red for both CE-BB and IR-ALADDINimages. **Figure S5.** The visualcomparison between contrast-enhanced black blood imaging and IR-ALADDINimaging. The mLVs are boxed in red for both CE-BB and IR-ALADDINimages. **Figure S6.** Thevisual comparison between contrast-enhanced black blood imaging and IR-ALADDINimaging. The mLVs are boxed in red for both CE-BB andIR-ALADDIN images. **Figure S7.** The visualcomparison between contrast-enhanced black blood imaging and IR-ALADDINimaging. The mLVs are boxed in red for both CE-BB and IR-ALADDINimages. **Figure S8.** The visualcomparison between contrast-enhanced black blood imaging and IR-ALADDINimaging. The mLVs are boxed in red for both CE-BB and IR-ALADDINimages.

## Data Availability

MRI data and the scripts for this research will be made available upon reasonable request to the corresponding author after permission by institutional review board.

## Abbreviations

ALADDIN: Alternate ascending/descending directional navigation
BB: Black blood
bSSFP: Balanced steady state free precession
CNS: Central nervous system
CSF: Cerebropinal fluid
FLAIR: Fuid attenuated inversion recovery
GM: Gray matter
IR: Inversion recovery
mLVs: Meningeal lymphatic vessels
PSC: Percent signal change
SSS: Superior sagittal sinus
TOF: Time-of-flight
