# Supplementary material for: Non-invasive flow mapping of parasagittal meningeal lymphatics using 2D interslice flow saturation MRI
Source: Fluids Barriers CNS. 2023 May 26;20:37. doi: 10.1186/s12987-023-00446-z (PMC10224581; doi:10.1186/s12987-023-00446-z)
Supplement: Supplementary file 1 — Additional file 1: Figure S1. IR-ALADDINsubtraction and baseline images across multiple slices from one representativevolunteer. Yellow arrowheads represent superior sagittalsinusand red arrows represent the meningeal lymphatic vessels. Figure S2. IR-ALADDINsubtraction images and baseline images from normal volunteers.Yellow arrowheads represent superior sagittal sinusand red arrowsrepresent the meningeal lymphatic vessels. Figure S3. IR-ALADDINimages of multiple measurements from the same subject to check therepeatability of IR-ALADDIN. Each red box is enlarged to the white box. Redboxes contain SSS in the A→Pdirection image and mLVs in the P→Adirection image. Figure S4. The visualcomparison between contrast-enhanced black blood imaging and IR-ALADDINimaging. The mLVs are boxed in red for both CE-BB and IR-ALADDINimages. Figure S5. The visualcomparison between contrast-enhanced black blood imaging and IR-ALADDINimaging. The mLVs are boxed in red for both CE-BB and IR-ALADDINimages. Figure S6. Thevisual comparison between contrast-enhanced black blood imaging and IR-ALADDINimaging. The mLVs are boxed in red for both CE-BB andIR-ALADDIN images. Figure S7. The visualcomparison between contrast-enhanced black blood imaging and IR-ALADDINimaging. The mLVs are boxed in red for both CE-BB and IR-ALADDINimages. Figure S8. The visualcomparison between contrast-enhanced black blood imaging and IR-ALADDINimaging. The mLVs are boxed in red for both CE-BB and IR-ALADDINimages. [file 12987_2023_446_MOESM1_ESM.docx]

Additional file Figures and Table for the manuscript

Non-invasive flow mapping of parasagittal meningeal lymphatics using 2D interslice flow saturation MRI

**Jun-Hee Kim MS1 | Roh-Eul Yoo2 | Seung-Hong Choi2 | Sung-Hong Park PhD1***


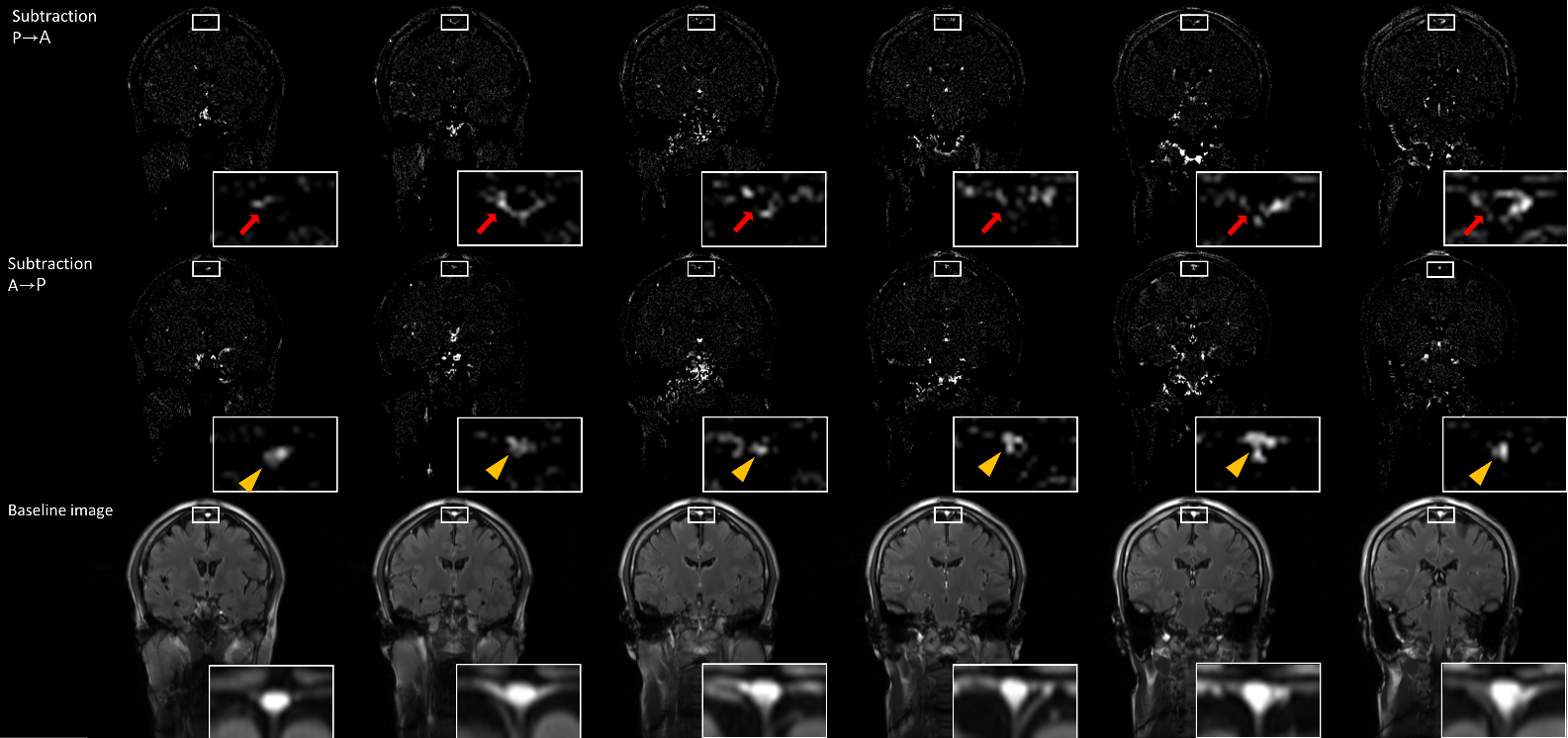


**Additional file F I G U R E S1 IR-ALADDIN subtraction and baseline images across multiple slices from one representative volunteer**. Yellow arrowheads represent superior sagittal sinus (SSS) and red arrows represent the meningeal lymphatic vessels (mLVs).


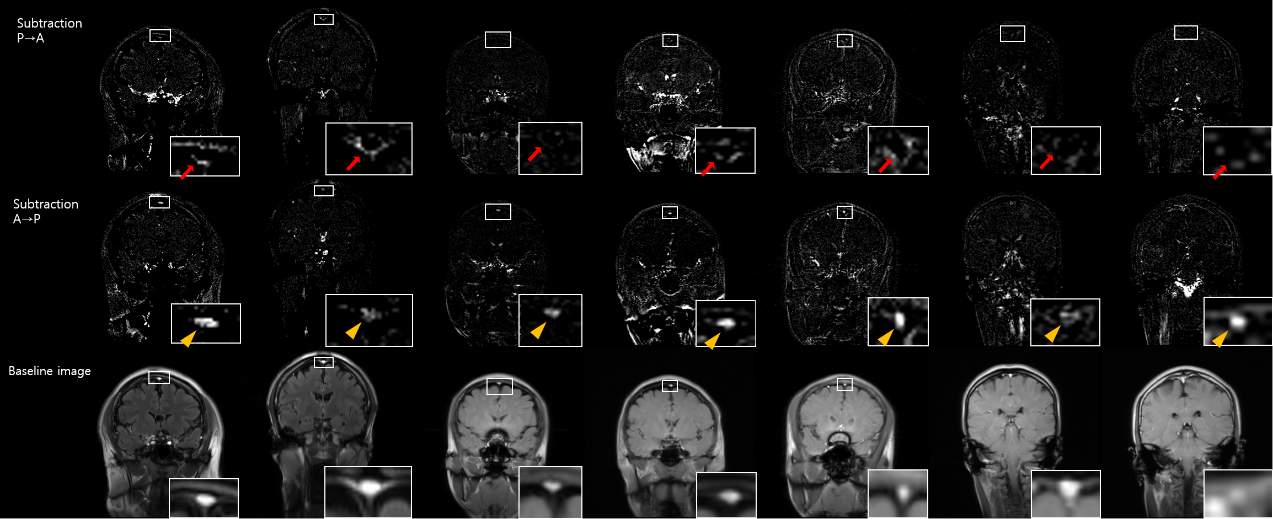


**Additional file F I G U R E S2 IR-ALADDIN subtraction images and baseline images from normal volunteers**. Yellow arrowheads represent superior sagittal sinus (SSS) and red arrows represent the meningeal lymphatic vessels (mLVs).


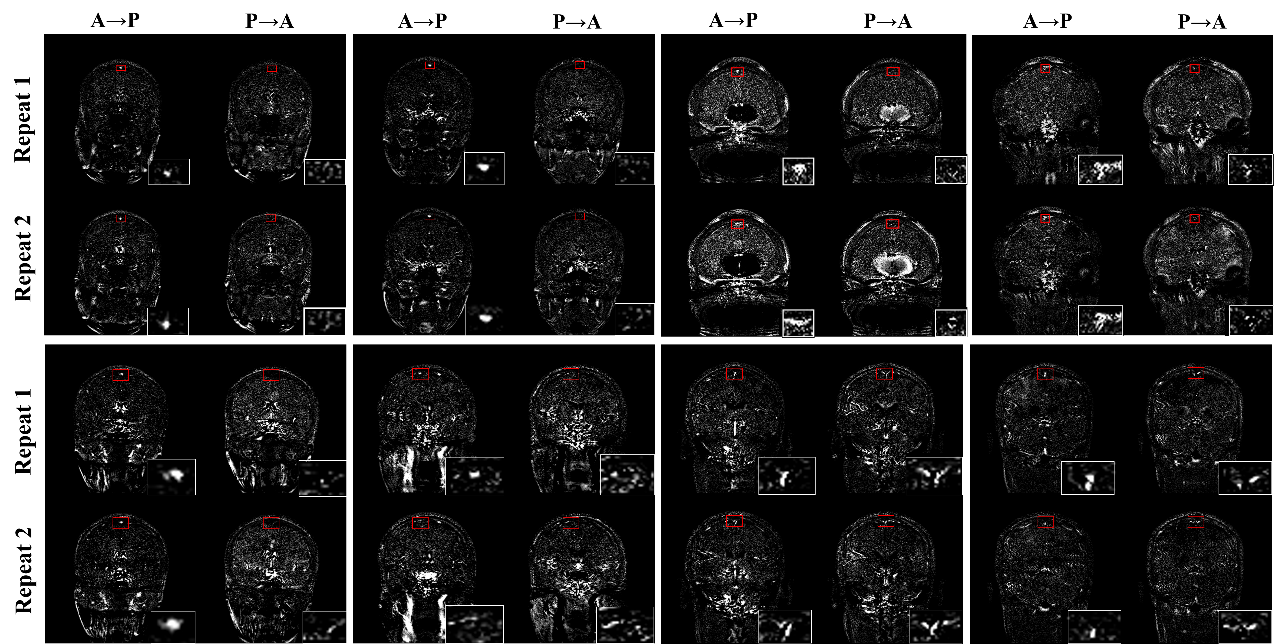


**Additional file F I G U R E S3 IR-ALADDIN images of multiple measurements from the same subject to check the repeatability of IR-ALADDIN**. Each red box is enlarged to the white box. Red boxes contain SSS in the A→P direction image and mLVs in the P→A direction image.


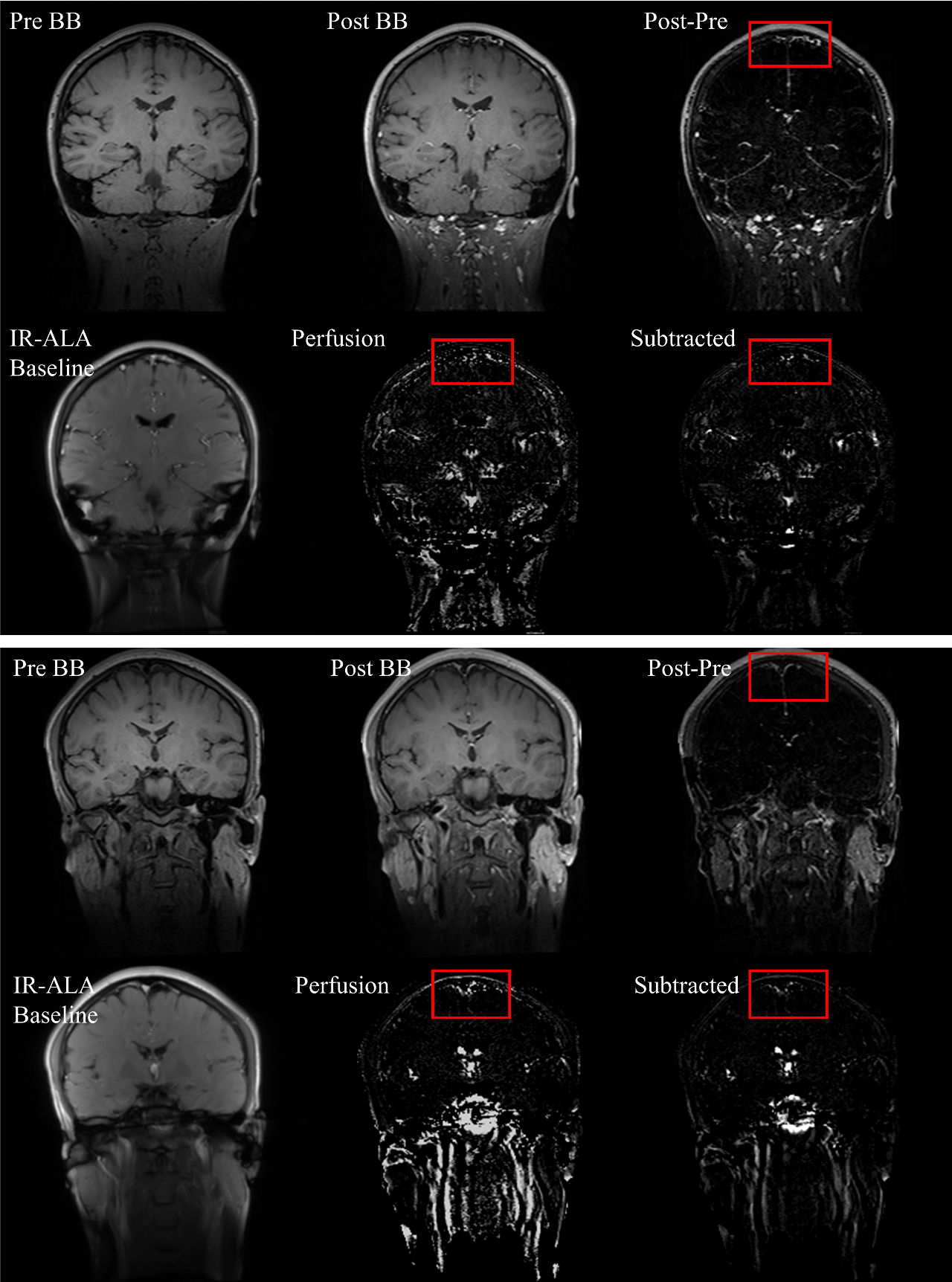


**Additional file F I G U R E S4 The visual comparison between contrast-enhanced black blood imaging and IR-ALADDIN imaging.** The mLVs are boxed in red for both CE-BB and IR-ALADDIN images.


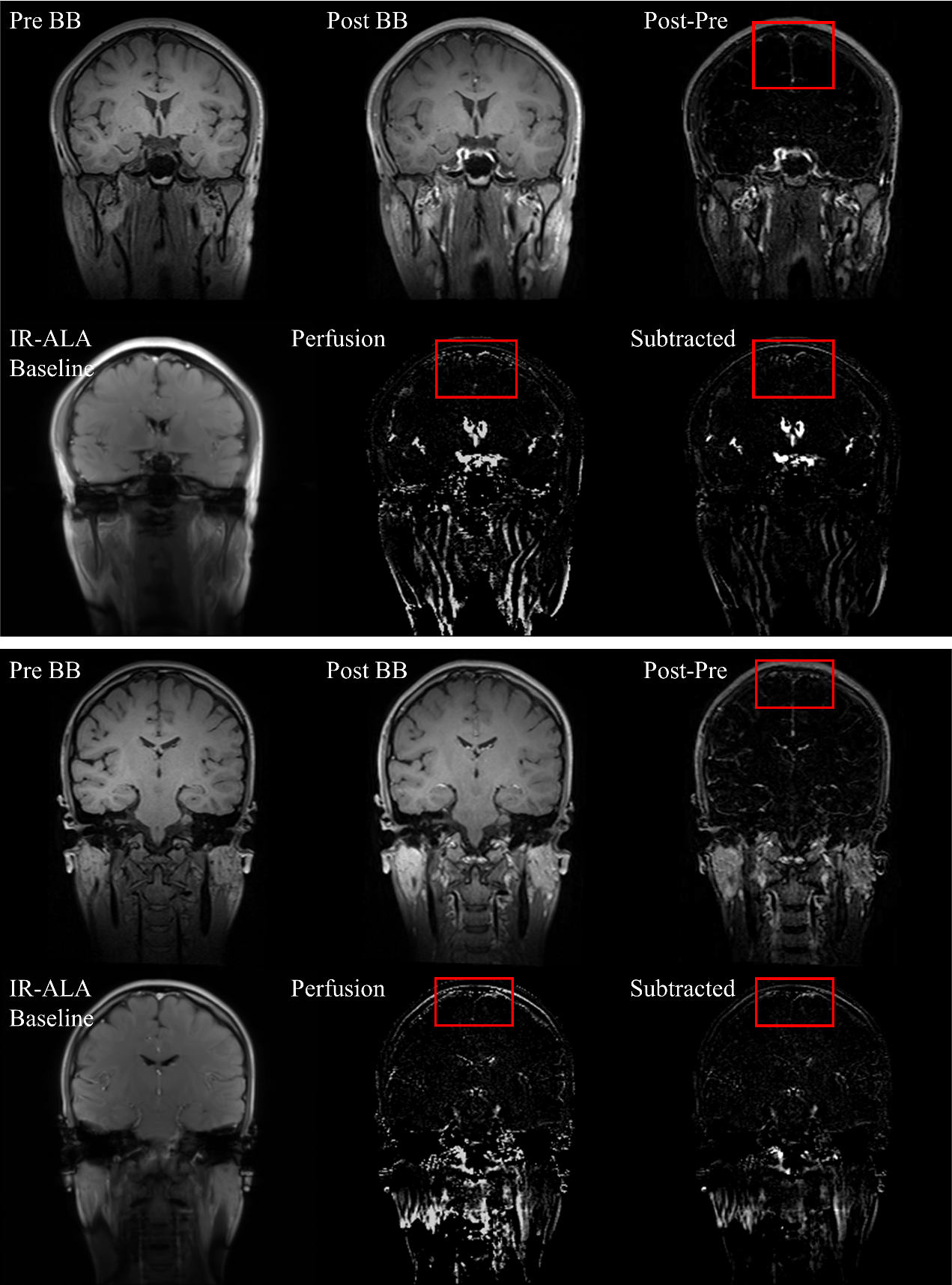


**Additional file F I G U R E S5 The visual comparison between contrast-enhanced black blood imaging and IR-ALADDIN imaging.** The mLVs are boxed in red for both CE-BB and IR-ALADDIN images.


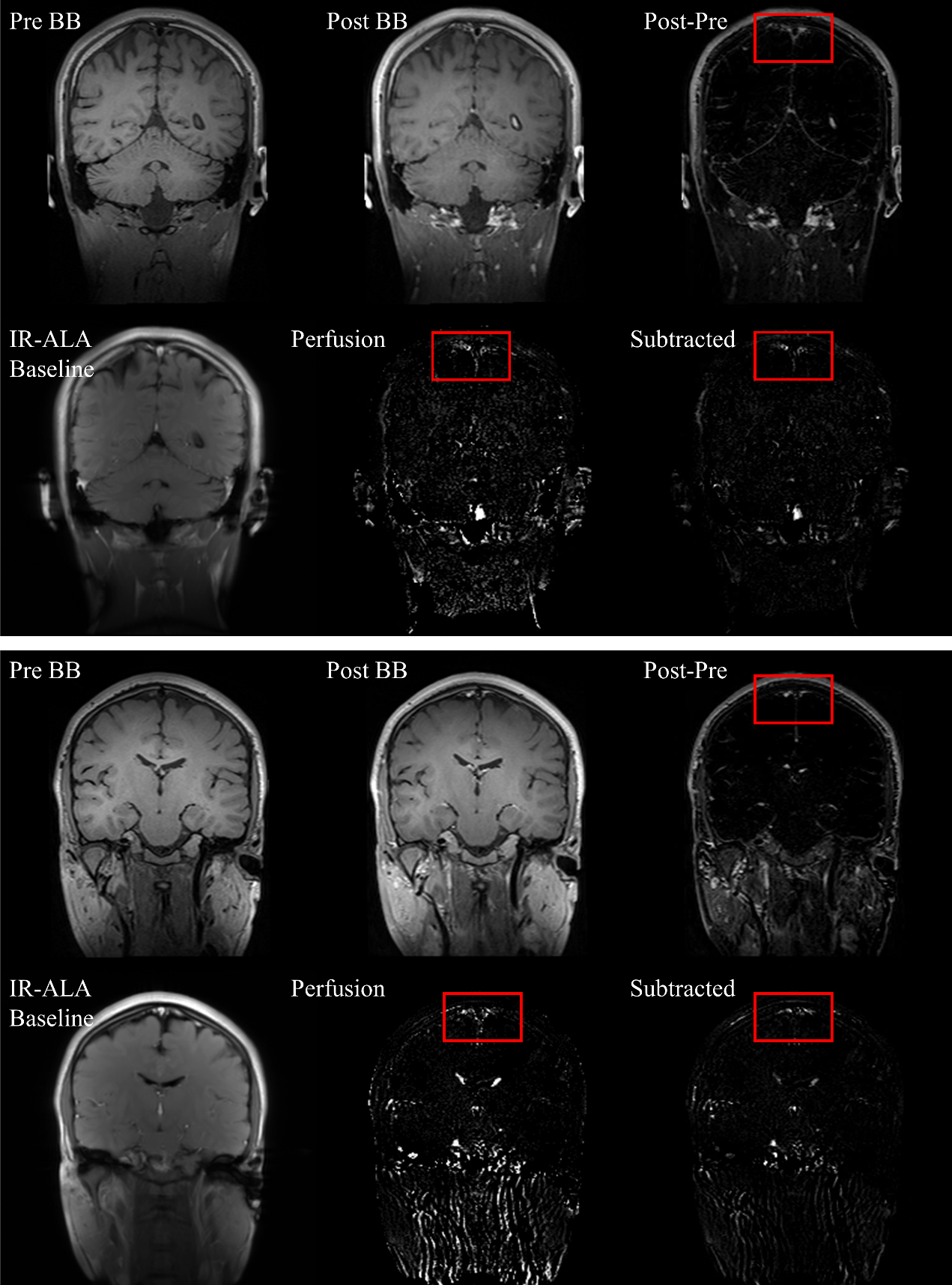


**Additional file F I G U R E S6 The visual comparison between contrast-enhanced black blood imaging and IR-ALADDIN imaging.** The mLVs are boxed in red for both CE-BB and IR-ALADDIN images.


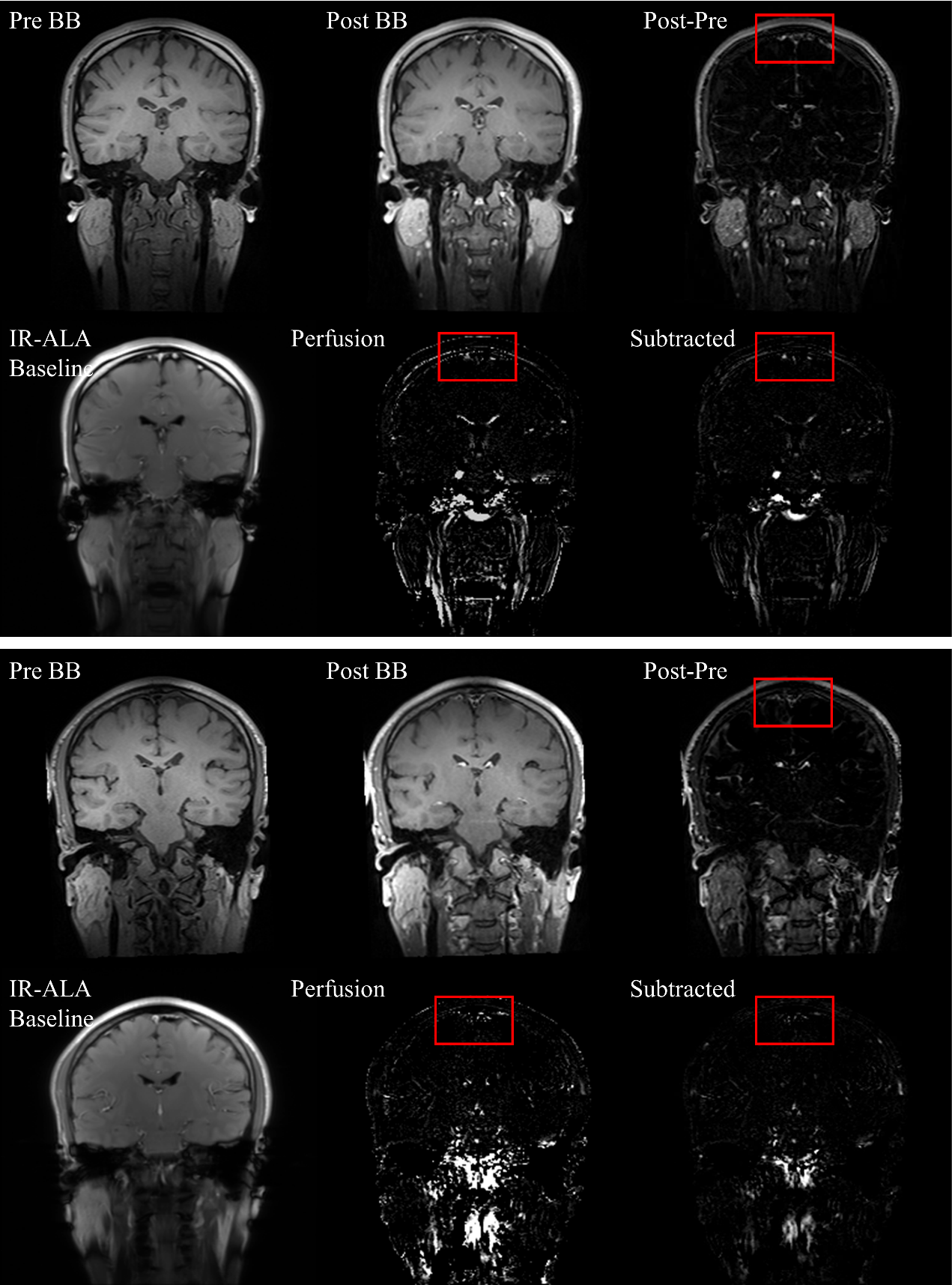


**Additional file F I G U R E S7 The visual comparison between contrast-enhanced black blood imaging and IR-ALADDIN imaging.** The mLVs are boxed in red for both CE-BB and IR-ALADDIN images.


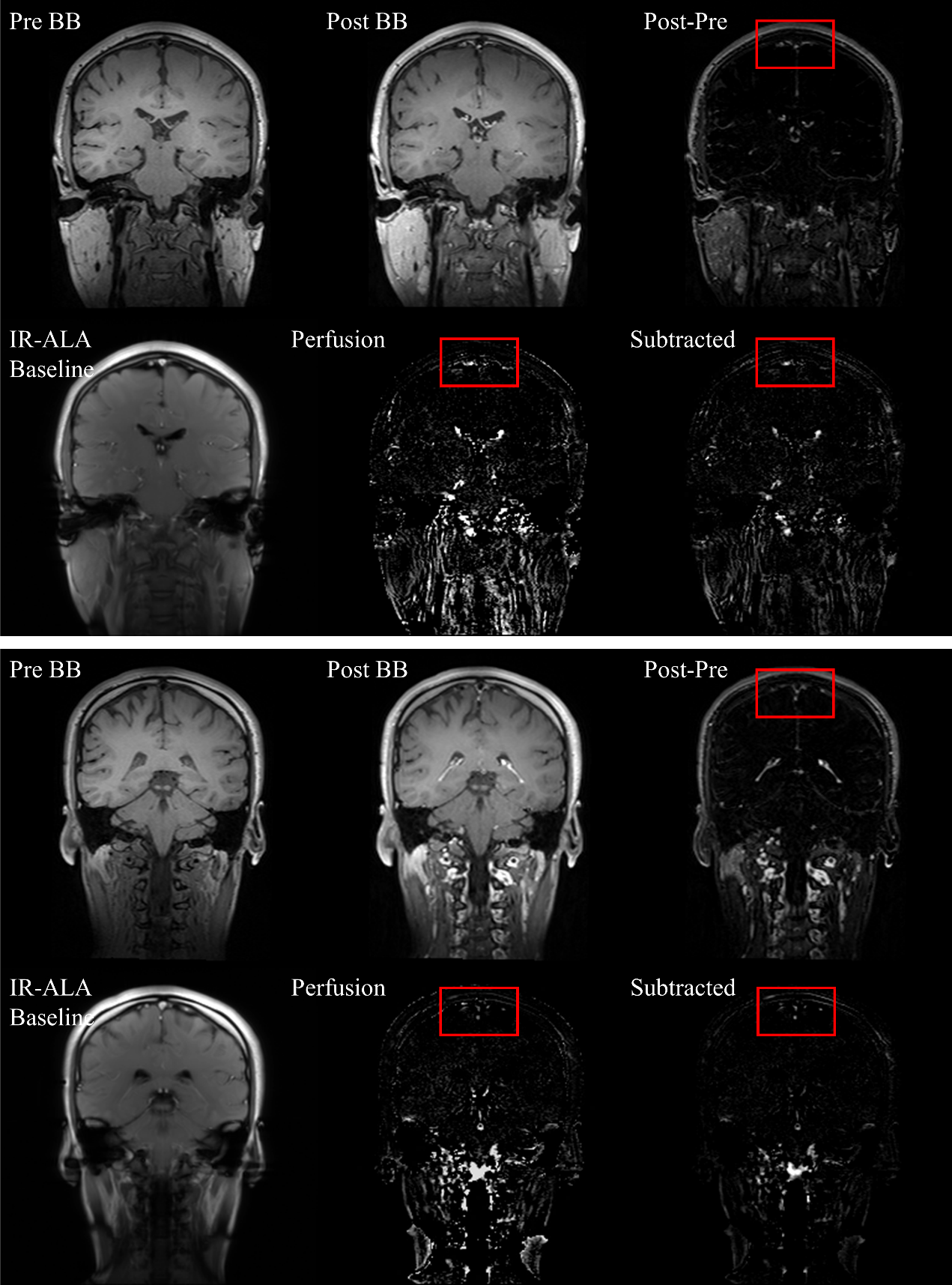


**Additional file F I G U R E S8 The visual comparison between contrast-enhanced black blood imaging and IR-ALADDIN imaging.** The mLVs are boxed in red for both CE-BB and IR-ALADDIN images.
